# Ultrasound Treatment of Buckwheat Grains Impacts Important Functional Properties of Resulting Flour

**DOI:** 10.3390/molecules25133012

**Published:** 2020-07-01

**Authors:** Joanna Harasym, Elena Satta, Urszula Kaim

**Affiliations:** 1 Adaptive Food Systems Accelerator–Science Centre, Wrocław University of Economics and Business, Komandorska 118/120, 53-345 Wrocław, Poland; urszula.kaim@ue.wroc.pl; 2 Department of Biotechnology and Food Analysis, Wrocław University of Economics and Business, Komandorska 118/120, 53-345 Wrocław, Poland; 3 Department of Agricultural and Food Science, University of Bologna, Piazza Goidanich 60, 47521 Cesena, Italy; elena.satta@studio.unibo.it

**Keywords:** grain, buckwheat, ultrasounds, grains, functional properties, pasting properties, DPPH, total polyphenols content

## Abstract

The benefit of not containing the gluten complex protein also provides problems with the achievement of typical and proper texture, especially in bakery products. Ultrasound (US) treatment has been previously studied on buckwheat as assistance treatment facilitating the release of antioxidant compounds. However, there is no study regarding the changes occurring in US-treated buckwheat grains regarding the structure-creating capacity, like water absorption, gelling, and pasting. The aim of this study is to the impact of US-treatment of buckwheat grains at 1:10, 1:5, and 1:2.5 solid:liquid ratio (in water). The particle size distribution, water absorption index (WAI), water solubility index (WSI), swelling power (SP), pasting characteristics, color, soluble, insoluble and total polyphenols content (SPC, IPC, TPC) and antioxidant activity (DPPH) were assessed in resulting flours. US-treatment caused specific agglomeration, resulting in bigger particles for 1:5, and 1:2.5 ratio treated samples, while higher dilution (1:10) increased smaller particle size fractions. The WAI and SP were the highest for the1:5 solid:liquid ratio sample, and the same sample revealed the highest peak viscosity, breakdown, and setback values. The ultrasound treatment increased the WSI, which was positively correlated with insoluble polyphenols content. The soluble polyphenols content decreased, and insoluble polyphenols content increased in all ultrasound treated samples. The DPPH scavenging activity remaining in grain after US treatment was lowered compared to the control sample. The relocation of pigments resulted in a redness and yellowish increase in all treated samples, while lightness was also increased but was most pronounced for a 1:10 ratio treated sample. The results suggest that ultrasound treatment of grain can improve the essential functional properties of buckwheat flour.

## 1. Introduction

The exploitation of ultrasounds as a form of energy transfer is rising in the food industry, especially in processing techniques susceptible to temperature, and ultrasonication is considered an emerging and promising processing method. The ultrasound-assisted treatment (UST) provides physical, chemical, and biochemical modifications in food matrix components via liquids (by cavitation effects), and gases (by acoustic pressure) contained [1]. The application of UST is growing, and its use focuses not only on mechanical operations like homogenization, mixing, degassing, extraction, filtration, crystallization or dehydration, but biological processes of fermentation, through its antifoaming actions, reduction in particle sizes, temporary or permanent modifications to viscosity, cell destruction and the dispersion of aggregates, inactivation of microorganisms and enzymes, or the sterilization of equipment, as well [2]. Currently, the UST results in a range of distinct benefits, like improving the techno-functional properties of food raw materials or enhancing the efficacy of processing conditions. The complexity of processed matrices is revealed by a whole spectrum of results obtained by UST, which is often very contrary due to different processing parameters like power, frequency, or the time of UST [3]. The main food macro-polymers like proteins, starch constituents, or fiber, respond specifically to UST.

Ultrasound application (frequency, sonication time), as well as pH, temperature, or ionic strength, affect the proteins’ physicochemical properties. The composition and structure of proteins have several functional properties that are fundamental to the quality characteristics of food. The absorption and release of water, the ability to form emulsions and foams, and the gelling properties depend on the proteins and can be modified by chemical or physical agents. High energy ultrasound can be considered an alternative to improve the protein solubility of different food sources. Various studies have reported an increase in protein solubility and a subsequent improvement in functional properties [4,5,6,7]. For the specific modification of food-derived proteins, the ultrasound processing is considered a standard and effective modification technology [8]. UST is also successfully exploited as an extraction assisting process [9,10,11]. The fast mass transfer has been observed during the ultrasound-assisted extraction of polyphenols and other antioxidant compounds from different matrix [12]. 

Buckwheat is gaining more and more attention as starch and protein-rich raw material, offering a substantial opportunity as functional food [13]. As a Polygonaceae family member, the plant and its fractions possess a high content of bioactive compounds, mainly of the polyphenols group [14]. Buckwheat can be stored for long periods without changing its chemical composition due to its high content of natural antioxidants, including tocopherols [15], phenolic acids [16], and flavonoids [17,18]. The seeds are rich in flavonols while the seedlings are rich in flavones. The main flavonoids found in buckwheat are rutin, orientin, vitexin, quercetin, isoorientin, and isovitexin [15,16,17,18,19]. In the seeds, only rutin and isovitexin can be found, while the others are contained in the hulls. Rutin (quercetin-3-rhamnosyglucoside) is a glycoside flavonol that can reduce capillary fragility associated with hypertensive disorders [18]. Several researchers were studying the amount [20,21] and characteristics [22,23] of antioxidants from buckwheat fractions, especially grains [24]. Both common buckwheat and Tartary buckwheat can serve as an excellent source of polyphenols compounds, and many studies explore the extraction of buckwheat grain with different solvents and techniques, primarily assisted by ultrasound [25]. Therefore, there are many results regarding both the quantitative and qualitative analysis of buckwheat polyphenols.

Buckwheat nutritional and phytochemicals content make it an excellent raw material for gluten-free applications [26]. Buckwheat grain nutritional characteristics resemble the typical cereals’ grain. The primary nutrients, like proteins, fat, and carbohydrates, are similar in proportions to those of the other cereals. The main difference is visible in the protein fraction, as buckwheat grain has the composition of amino acids more balanced; therefore, its nutritional value is higher. The percentage of the essential amino acids is very similar in comparison to the other cereals, following the FAO recommendations. The limiting amino acid in the buckwheat is the isoleucine, while it is the lysine for the other cereals. Buckwheat proteins are rich in lysine (5.5–6.1%) and contain less glutamic acid and proline, but more arginine and aspartic acid, than the proteins generally found in cereals. About 56% of glutamic acid and aspartic acid is in the form of amides. The correlation between the amount of basic, neutral, or acid amino acids is negative. Two rare amino acids, L-2-(2-furoyl) alanine, a neutral amino acid, and fagonine, a non-protein amino acid, have also been identified [27]. The main component of buckwheat grain is starch (over 70% of its dry basis) [28], and because of this, the quality of buckwheat food products is strongly dependent on the starch structure and functional properties [28,29]. Ikeda et al. [28] showed that starch and amylose contents were highly correlated with the springiness of heated buckwheat dough, probably due to the gelling capacity of amylose and starch. Buckwheat starch, therefore, is useful as a fat replacer and extruded product ingredient [30]. 

Ultrasound-assisted treatment of buckwheat flour is widely recognized as a process supporting the antioxidant extraction, and many studies optimized its yield. The remaining antioxidant potential of raw material can be sharply decreased as ultrasound-assisted extraction of polyphenols is a very effective treatment. However, there is scarce information about the antioxidant activity left in buckwheat grain/flour after the treatment. Additionally, there are also no data regarding the functional properties of remaining after UST grain/flour residues. 

Therefore, the objective of this study was to determine if the ultrasound treatment of intact buckwheat grain changes the essential functional characteristics of buckwheat flour obtained from such treated grains.

## 2. Results and Discussion

### 2.1. Granulometry of Flours From Ultrasound Treated Grains

Buckwheat (*Fagopyrum esculentum* Moench) grain of the Kora variety was stone-disk-milled and the resulting flour content was as follows: the initial water content was 11%, protein 13.2%, fat 3.13%, dietary fiber 4.82%, ash < 2% and carbohydrates 83%, of which starch content was about 65%. The flour granulometry measured by sieving is shown in Table 1.

The flours’ fraction distribution among all the samples differs according to particle size and treatment. All samples were sieved through a 500 µm sieve. Sample characterized with the highest number of fractions with particles above 200 µm were samples treated with 1:2.5 and 1:5 ratio; although the difference was statistically insignificant from the control sample, the trend was observed. Meanwhile, a sample treated with a 1:10 ratio shown the lowest level of >200 µm fraction. The more uniformity was observed for all samples regarding the >150 µm fraction, except the 1:10 ratio-treated one, which showed almost twice the lower level of this fraction. Oppositely for >106 µm particle size fraction level, the highest amount was observed in a 1:10 ratio-treated sample, while 1:2.5 and 1:5 ratios treated samples contain the lowest levels of this fraction. The smallest particle size fraction, whose particles were below 106 µm, was the most abundant in a 1:10 ratio-treated sample, while the rest were not significantly different from control sample granulometry. 

Jambrak et al. 2014 [31] treated whey protein isolate and whey protein concentrate with different frequencies and times. For 40 kHz ultrasound frequency, 15 min exposure time, about 28 °C and 1:10 solid:liquid ratio, they observed the general trend of decreasing both the particle size of 90% of fraction in a sample and 10% of fraction in WPI sample after ultrasound treatment vs. control one. However, the distribution changed as for 50% of treated samples; the average particle size was increased, being the highest among all the treated samples, including control. The WPC, which is not so enriched in protein, revealed the well-known behavior of decreasing the particle sizes after sonication. Authors discuss that ultrasound can induce structural changes in proteins, which are more due to breaking the intermolecular hydrophobic interactions (weak), rather than the cleavage of strong bonds like peptide or disulfide linkages. However, the particles subjected to shear stress may undergo the raised speed of collision, resulting in increased aggregation. Ding et al. 2019 [32] treated 10% suspensions of resistant starch (RS3) using 20 kHz, 30 min treatment of 2 s on/2 s off cycles and 100 W of power, and observed that the D [3,4], D [2,3] and d_0.5_ of treated RS3 sample increased, which means that large mean diameters, both on a volumetric and surface basis, were enlarged. However, the specific surface area was lowered. The authors claim that such results confirmed the observation they made with scanning electron microscopy and prove that ultrasonic treatment could facilitate the reaggregation of RS3 granules to form large particle size. Both observed phenomena impact the granulometric characteristic obtained during our experiment. The modification of particle size fractions in the grain is far more complicated than in isolated biopolymers like proteins or starch. The main components of grain are carbohydrates and protein, but of carbohydrates, not only starch changes after ultrasound treatment but also dietary fiber. Hu et al., 2019, modified the soluble and insoluble dietary fiber (IDF) water-extracted from sunflower meal and observed that the particle size of IDF decreased, and the distribution was concentrated [33]. Fraction >200 µm was strongly negatively correlated (Pearson correlation) with >106 µm fraction (−0.967; *p* ≤ 0.01), while >150 µm was strongly negatively correlated with <106 µm fraction (−0.963; *p* ≤ 0.01).

### 2.2. Gelling Capacities of Flour after Ultrasound Treatment

Ultrasound as a form of mechanical energy transfer can contribute to the physical modification of the plant matrix exposed to them. Such modifications in the starchy grain can be assessed based on the observation of flour’s in-water behavior. The hydration properties of flours, as well as their response to mixing, heating, and centrifugation, reveal substantial information about the internal characteristics of particular compounds of flour, and especially the changes eventually introduced by ultrasonication. The impact of the ultrasound treatment on hydration characteristics is presented in Table 2.

In all the treated samples, the water absorption index (WAI) was lowered significantly vs. the control sample, and the same behavior was observed for swelling power (SP). Water solubility index—the quantity marker of soluble in water compounds—increased significantly in all the treated samples vs. the control one. The lowest value of water absorbed and maintained in the form of gel (WAI) was observed for flour resulting from grain treatment at a solid:liquid ratio of 1:10, and swelling power (SP) was also the lowest for that sample. The highest value of WAI was in sample resulting from buckwheat grain treated at a ratio of 1:5; similarly, for that sample, the highest SP was noted. The WAI and SP values of this sample most resemble the control sample characteristics. The untreated sample was also characterized by the lowest water solubility index (WSI). The ultrasound treatment in the conditions of the lowest solid:liquid ratio, 1:2.5, resulted in moderate changes in hydration parameters.

The water absorption index reflects the capacity of a sample to absorb and maintain water by forming a gel in the cooling–heating cycle and then the gel resistance to release water during centrifugation. Such behavior is strongly related to starch characteristics, especially the amorphous and crystalline domains in starch granules and interactions between them. After heating over the starch gelatinization temperature, the starch granules swell and rupture, releasing amylose. Solubilized amylopectin fragments and amylose chains, once leached to the solution, reorganize and align themselves during cooling to form a gel structure. The degree of starch granule rupture, amylose leaching level, and partitioning of amylopectin result in a different capacity of the sample to form a gel of a specific syneresis resistance. Lowering WAI can be associated with starch granule organization disordering as the effect of energy dissipation transmitted by ultrasounds. Mechanical waves crossing the internal structure of hydrated grain can lose the starch grain structure, leading to a higher disintegration level after milling. Yu et al. 2018 compared milled buckwheat grain using different mills, and the WAI of flour obtained from stone disc mill was similar to our control sample [34]. However, the sample from the high-speed universal grinder (26,000 rpm) showed WAI values comparable with our ultrasound treated samples. The water solubility index (WSI) is a parameter that reflects the number of compounds released from the gel. Usually, it can be used as a biopolymer degradation marker and an indicator of the dextrinization degree of starch. However, there was no significant difference in WSI among all treated samples, which suggests that changes resulting in WAI and SP lowering were more due to the partial partitioning of starch than strong depolymerization, which otherwise would increase the content of the soluble compounds and WSI values. A similar observation was made by Yu et al. 2018 who observed the highest WSI for the sample of buckwheat flour obtained by high-speed universal grinder milling, while stone disc mill resulted in a lower WSI value [34]. The higher WSI value can also confirm the starch fragmentation by ultrasound in the US-treated samples since a previous study positively correlated WSI with damaged starch. 

Li et al. 2014 analyzed buckwheat starch physically modified and observed a significant increase in soluble solids after treatments like micronization and pre-gelatinization, even in samples of isolated starch [35]. Zhu and Li et al. 2019 treated quinoa flour with ultrasounds in a solid:liquid proportion 1:20 for 1.2, 2.4, 4.8, 9.6, and 19.2 h and observed an increase in WSI with prolonged ultrasound exposure while SP was decreasing [36]. The lack of difference in soluble solids among treated samples suggests that specific amounts of compounds were dissolved and liberated to process liquid from a buckwheat grain during ultrasound treatment, which resulted in insignificant differences among WSI.

### 2.3. Pasting Properties Characteristics

Evaluation of pasting viscosity parameters is important to categorize the raw material for end product recommendation. Table 3 presents the main pasting data of ultrasonicated buckwheat samples.

The significant difference was observed in peak viscosity (PV) only for the sample treated with a 1:5 solid:liquid ratio; the rest were not significantly different from the control sample. The hold viscosity (HV) was the lowest for samples treated at ratio 1:10 and ratio 1:2.5) and significantly different from both control and ratio 1:5 samples. The same characteristics were observed for the final viscosity value (FV). The lowest breakdown viscosity was recorded for the control sample, while the lowest setback viscosity revealed the sample treated at 1:10. The highest results for breakdown viscosity and setback viscosity were observed for the sample treated at 1:5. 

Pasting peak viscosity value reflects the water-binding capacity of flour, which is the result of combined starch, protein, and fiber water-binding capacities. Ultrasound is capable of modifying starch [37], protein [38], and fibrous matter [39], and the level of modification depends on the ultrasound frequency, duration, temperature, the moisture content of the system. The ratio 1:5 seems to individually modify the internal structure and starch-protein interaction, providing higher peak viscosity than the control sample. Fitzgerald et al. 2003 proved that the proteins and specific botanic characteristics of starch could significantly change the pasting profile, especially peak viscosity [40].

Yu at al. 2018 after wet milling and sieving (90 mesh) of buckwheat, obtained higher pasting viscosity, which suggests that segmentation of grain endosperm occurred during US treatment [34]. Hold viscosity value was lowered for samples treated with a 1:10 and 1:2.5 ratio, and such treatment improved the ability of the sample to withstand the conditions of 95 °C heating and shear stress. Such resistance is essential in many industrial processes. Zhu et al. 2019 processed quinoa flour with ultrasound and observed similar results of lowering the HV. This suggests that appropriate ultrasound treatment can be an effective method for modulation of the heat and shear resistance of buckwheat pastes [36]. The same behavior was observed for final viscosity, as the only sample treated at ratio 1:5 was able to maintain the same final viscosity as a control sample. In two other treated samples, FV was lowered. Usually, in the cooling period, the viscosity increases and stabilizes to a certain degree, which depends on re-association between mainly amylose chains liberated in the heating period. The lowering of FV in some treated samples can be caused by amylose leaching into the water during US treatment, which resulted in its lower amount in remaining flour. Zhu et al. 2019 also observed the lowering of FV for ultrasonicated quinoa flour [36]. Final viscosity is a critical quality parameter that indicates the ability of the tested sample to form a viscous paste or gel after heating and cooling. 

Breakdown viscosity values reflect the degree of granule disruption after reaching maximum viscosity value. The significant rise in breakdown was observed for all treated samples, while the 1:5 ratio-treated sample reflected the 450% rise of breakdown vs. control sample. A similar massive increase in breakdown (625%) in flour from sonicated navy bean was observed by Ghafoor et al. 2014 [41]. The susceptibility to disruption is attributed to the botanical origin of starch, the starch granule size, and the level of damage resulting from the previous treatment. The degree of viscosity breakdown also depends on other viscosity contributors as proteins and fibers. The setback was either decreased in the sample treated at ratio 1:10 or increased in the sample treated at ratio 1:5. Ghafoor et al. 2014 [41] and Zhu et al. 2019 [36] also observed a rise in setback viscosity in the sonicated navy bean flour and quinoa flour, respectively, however, with a prolonged time of ultrasound treatment, the setback was decreased [36].

There are several other stages during pasting that notify the changes occurring in the heated matrix. Some of them are presented in Table 4.

The highest viscosity at 1 min was noted for the control sample, while both samples 1:10 and 1:2.5 showed the lowest viscosity. Viscosity measured at 1 min reflects the moment when all the starch granules remain intact and hydrated but are not ruptured. The highest value for the control sample confirms that ultrasound treatment disrupts partially starch granules and facilitates amylose leaching. Other studies reported that damaged starch could restrict the swelling of starch granules [42]. There was no statistically significant difference between pasting point viscosity in all samples, including control. Pasting point time was the shortest for the sample treated in a solid:liquid ratio of 1:5, while the longest was for the sample treated in solid:liquid ratio of 1:10, and all the samples revealed significant differences in this parameter. The same characteristic was observed for pasting point temperature. 

Pasting point viscosity and pasting point time are two indicators of the starting moment of the pasting phenomenon. As the temperature increases, the starch granules continue to swell and eventually rupture. Amylose chains are leached into solution followed at a slower rate by the amylopectin chains. Under the mechanical shear applied by mixing, the polymers tend to align themselves, increasing the viscosity in a balanced way with granule swelling. Pasting temperature is the temperature at which the viscosity begins to increase during the heating process, while a low pasting temperature indicates a lower resistance to swelling and rupture.

Pearson correlation among functional parameters revealed that WAI is strongly positively correlated with a viscosity at 1 min (0.955;*) and SP (0.996;**), while WSI is strongly positively correlated with hold time (0.953;*). The strong positive correlation was also observed between final viscosity and setback (0.961;*) as well as pasting point and >106 µm fraction (0.972;*). 

### 2.4. Soluble, Insoluble and Total Polyphenols Content in Flours after Grain Ultrasonication

**Figure 1:**
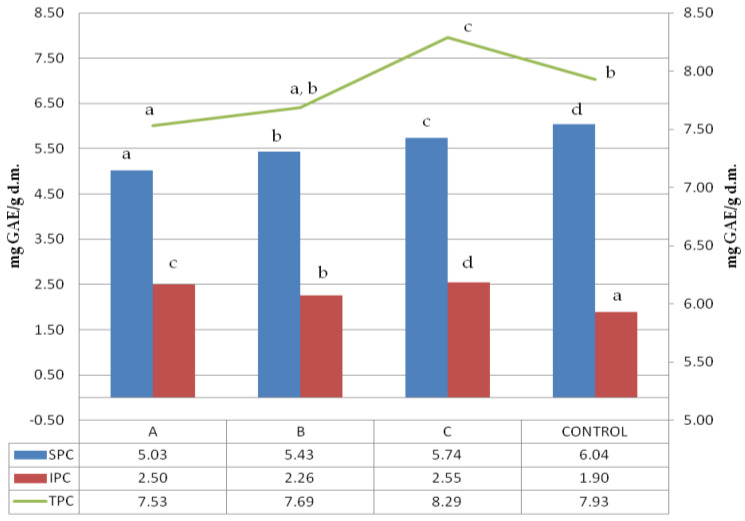
Soluble, insoluble and total polyphenols content. 1:2.5, 1:5, 1:10-solid:liquid ratio of buckwheat grain samples ultrasound treated in water; control–sample of untreated buckwheat grain. Lower-case letters mean values significantly different at *p* ≤ 0.05 in the particular data series (SPC, IPC, TPC). SPC-soluble polyphenols content, IPC-insoluble polyphenols content, TPC-total polyphenols content.

Folin–Ciocalteu reagent can detect mainly phenol rings, which are usually associated with polyphenol presence. The soluble, insoluble and total polyphenol content (SPC, IPC, and TPC) of ultrasonicated samples are shown in Figure 1.

The control sample was characterized by the highest number of soluble polyphenols; however, the untreated buckwheat did not have the highest total polyphenol content. A clear dependence of the soluble polyphenols extracted from the sample on the solid:liquid (s:l) ratio was observed, revealing that the higher the s:l ratio of initial ultrasound treatment, the lower the soluble polyphenol content recorded. A surprising trend was observed in insoluble polyphenols where the highest amount was obtained for samples treated in the lowest solid:liquid ratio of 1:2.5. As the loss of soluble polyphenols was the lowest, the increase in insoluble polyphenols was the highest. However, there was no such trend for other samples, like the one treated at a 1:5 solid:liquid ratio, which revealed the lowest insoluble polyphenol content. The significant difference observed among total polyphenol content within the treated samples referred to samples treated at 1:10 and 1:2.5, with both significantly different between themselves and also compared to the control.

The lowest TPC was noted for the sample treated with the highest dilution (1:10). The sample treated with a 1:5 solid:liquid ratio showed particular characteristics because TPC was not significantly different either from 1:10 or control sample. All the samples revealed a significant variation in results, suggesting the complex nature of the phenomena that occurred during ultrasound treatment. Dzah et al. 2019 exposed Tartary buckwheat to ultrasonication in different solid:liquid ratios and observed that ratio was positively correlated with SPC [25]. In our research, we have measured the soluble polyphenols in solids remaining after extraction. We observed that in the sample treated with the highest solid:liquid ratio, was the lowest soluble polyphenol content, which confirms the trend observed by Dzah et al. 2019 [25]. Zhu and Li 2019 treated quinoa with ultrasound and observed the lowering of the SPC vs. control sample, which is in agreement with our observations [36]. Insoluble polyphenols’ content was strongly positively correlated with WSI (0.97;*), which was probably dependent on the solubilization of soluble residues during the first stage of polyphenol extraction, and hence the concentration of compounds containing insoluble polyphenols. 

### 2.5. Antioxidant Activity Changes as Result of Treatment

The colorimetric assay vs. 2,2-diphenyl-1-picrylhydrazyl, used as a typical method of radicals scavenging activity measurement is considered to be mainly based on an electron transfer reaction, while hydrogen-atom abstraction is a marginal reaction pathway. Such scavenging activity strongly depends on compounds extracted from the analyzed sample, while different solvent mixtures have distinct extractive power. The extracts were made using two different polarity solvents: acidified methanol of 88% (SP) and subsequent extraction by 50% methanol, followed by 50% acetone (DPPH). The results are presented in Table 5.

The control samples had the highest antioxidant activity per d.m. regardless of the solvent used, and the same characteristic regarding solvent was observed for all samples. Two-factor ANOVA showed that the impact of treatment was statistically significant at the level of *p* ≤ 0.001, while the effect of solvent could be neglected. However, the strong second-order interaction was observed for *p* ≤ 0.001 between two factors as the solvent used modified the activity vs. DPPH obtained from ultrasound treatments in different solid:liquid ratios. 

The polarity of two used solvents is quite different, as acidified methanol is less polar than 50% methanol and 50% acetone (polarity index of methanol and acetone is 5.1, and water is 9.0). It seems that compounds of antioxidant capacity vs. DPPH were of mixed polarity. The highest activity among treated samples was obtained for samples extracted from grain treated in a ratio of 1:5 with 50% methanol and 50% acetone. 

Samples obtained with acidified methanol were not significantly different among all treated samples, while a sample from treatment 1:2.5 revealed the lowest activity in extracts made with 50% methanol and 50% acetone. Xu et al. 2019 have treated green tea with ultrasound and the observed higher DPPH scavenging percentage, which is in agreement with our results, showing a lowering in DPPH radical scavenging activity in remaining flour [10]. 

Zhu at al. 2019 ultrasonicated quinoa flour and measured the DPPH as μmol TE and observed a lowering in radical scavenging activity after sonication in flours [36]. Xia et al. 2020 had studied the impact of the ultrasonication of DPPH radical scavenging activity in wholegrain rice and observed a significant lowering of EC_50_ value in grain [43] which means that many antioxidant compounds were liberated in wholegrain after ultrasound treatment, or some of them were neutralized with free radicals appearing as a result of cavitation phenomena in liquids. 

Buckwheat grain after ultrasound irradiation showed varied responses in different parameters. The antioxidant activity vs. DPPH is the typical reaction of the different natural compounds present in plants. Dzah et al. 2019 have studied both total extracted phenols and antioxidant activity of extracts obtained by ultrasonication from Tartary buckwheat and confirmed that where the higher DPPH was observed, the lower IPC was observed [25]. A similar correlation was observed in our study.

### 2.6. Color Changes after Ultrasound Treatment 

Color is an essential parameter of each food ingredient; usually, its change is connected with pigments’ presence or chemical modification of existing ingredients. The color changes in buckwheat flour resulting from ultrasound treatment of grains are shown in Table 6. 

The control sample had the highest lightness among all treated samples and the lowest reddish and yellowish color. Chroma was the lowest for control, while hue was the highest. The intensity of yellow color was changed slightly with the treatment, but a significantly different value was noted for the sample treated with a 1:10 solid:liquid ratio vs. control. That sample also had the highest chroma and the lowest hue, being the most reddish of all samples. The color changes were not linearly correlated with the increase in solid:liquid ratio. The color parameters of flour are important attributes affecting their application. According to results a great increase (*p* ≤ 0.05) in the values of a* and b*, as well as a decrease in the value of L*, were observed after ultrasound exposure, suggesting the simultaneous enhancement of color parameters for red and yellow and darkening, respectively. The darkening of colors of ultrasonicated flours could be due to the transport of pigments from outer layers occurring as an effect of the etched bran layer. After sonication in water, the soluble pigments, mainly red carotenoids and yellow xanthophyll pigments from bran, were released into endosperm of the grain. The L* parameter lowering could be due to reduced sugar liberation due to partial starch polymers partitioning and a Maillard reaction during drying. Similar results for L* lowering were observed by Ding et al. 2017 after the ultrasound treatment of rice flour [44]. Xia et al. 2020, while treating the whole grain rice, observed a simultaneous reduction in color parameters for red, yellow, and darkness, respectively [43]. The authors suggest that color changes were due to the transfer of colored components into the aqueous environment during incubation. 

Pearson correlation was found among antioxidant values of high polarity extracts and all the color parameters as follows: L* (0.963;*), a*(−0.965;*0, b* (−0.961;*), C (−0.954;*) and h (0.962;*) proving the essential contribution of pigments into antioxidant activity. DPPH was strongly correlated with all color parameters positively with L *(0.95;*) and hue (0.98;*) and negatively with a (−0.99;*), b (−0.97;*) and chroma (−0.97;*).

## 3. Materials and Methods 

### 3.1. Materials

The buckwheat grain of Kora variety was kindly provided by Grupa Producentów Ekologicznych Dolina Gryki sp. z o.o. (Międzylesie, Poland). The grain was previously mechanically dehulled by the manufacturer and provided in 20 kg bags. All reagents were of analytical grade and for dilutions and treatments the distilled water was used.

### 3.2. Grain Ultrasound Treatment 

Ultrasound exposition proceeded at 5 L capacity ultrasound bath (WAH LUEN ELECTRONICS, Shantou, China) equipped with two transducers of 45 kHz working frequency each. The treatment lasted for 15 min and was provided at a maximum power of 100 W. Samples of buckwheat grain were exposed in distilled water using different solid:liquid ratio as follows—1:10, 1:5, and 1:2.5. Precisely, 250, 500, and 1000 g of buckwheat grains were weighed, respectively, and immersed in the treatment tank containing 2.5 L of distilled water. The even exposure of grains to ultrasounds during processing was supported by gentle agitation with two mechanical stirrers (RW20, Janke and Kunkel, Staufen, Germany) at a speed of 90 rpm. After treatment, the grain samples were soaked on the drainer, gently drained with a paper towel, and dried at 50 °C in the laboratory oven (SML, Zalmed, Łomianki, Poland) for 24 h.

### 3.3. Proximate Content Measurement

The average moisture content of samples was assessed according to Official Method AACC 44-19 (AACC, 2000)-Moisture-Air-Oven Method, Drying at 135 °C. Ash content was determined by incineration method, lipid content was measured by gravimetric method using a Soxhlet apparatus with hexane as extraction solvent, and total protein content (*N* × 5.7) was analyzed by titrimetric method using a Kjeldahl distillation unit. Starch content was measured with the total starch method using Megazyme Kit (Megazyme LTD, Bray, Ireland).

### 3.4. Flour Preparation

Dried grains were milled on a stone disc mill (Fidibus21, KoMo, Hopfgarten, Germany) and sieved on the 500 mm sieve (CISA, Bracelona, Spain) using a sieve shaker (LAB-11-200/UP, EKO-LAB, Wałbrzych, Poland).

### 3.5. Granulometric Analysis

The product samples were sieved with the vibratory sieve shaker LPzE-2e (Multiserw Morek, Brzeźnica, Poland) at 0.65 mm vibration amplitude for 10 min with screens of 80, 106, 125, 150, 180 and 200 microns. Due to a low quantity, some fractions were joint, resulting in four fractions of >200, >150, >106 and <106 µm.

### 3.6. Gelling Properties

The gelling properties of flour were assessed as water absorption index (WAI), water solubility index (WSI) and swelling power (SP), which were also determined as technological functional properties of all the buckwheat samples with the method suggested by Kaushal et al. 2012 [45] with minor modifications by Abebe et al. 2015 [46]. A 3.00 g sample was effectively dispersed in 30 mL of distilled water and heated at 90 °C in the water bath (MLL147, AJL Electronics, Kraków, Poland) for 10 min. Then, the samples were cooled down to room temperature and centrifuged at 4000× *g* for 10 min (MPW-350, MPW, Warszawa, Poland). The supernatant was poured into a pre-weighed stainless steel Petri dishes to determine the solid content and left for total evaporation at 110 ºC in the laboratory oven (SML, Zalmed, Łomianki, Poland) for 24 h. The sediment after centrifugation was weighed, and the weight of dry solids was recovered, after evaporation of the supernatant. All the values were taken in triplicate. WAI, WSI and SP were calculated from the following equations

WAI = W_H_/W_S_ [g/g d.b.], (1)

WSI = W_SL_/W_S_ × 100 [g/100 g d.b.] (2)

SP = W_H_/W_S_ − W_SL_ [g/g d.b.] (3)

where W_H_ is the weight of the hydrated sample after heating and centrifugation, and W_S_ the initial weight of the sample, W_SL_ is the weight of solids after evaporation. The results were expressed as grams of water absorbed per grams of dry basis.

### 3.7. Pasting Properties

The pasting properties of the sample were determined with Rapid Visco Analyser (StarchMaster2, Newport Scientific, Sydney, Australia) according to using ICC Standard method 162. Briefly, the temperature profile used was as follows: holding at 50 °C for 2 min; a heating-ramp from 50 to 95 °C at a rate of 5 °C/min; holding at 95 °C for 5 min; then cooling back to 50 °C at a rate of 5 °C/min, and finally holding at 50 °C for 4 min. The peak viscosity (PV), hold viscosity (TV), final viscosity (FV), breakdown (BD), and setback (SB) were obtained as main pasting parameters. Additionally, pasting point viscosity (PPV), pasting point time (PPT), pasting point temperature (PPTp), viscosity at 1 min (V @ 1 min), hold time (HT) and hold temperature (HTp) were recorded. 

### 3.8. Polyphenols Content

The soluble, insoluble, and total polyphenols content was assessed according to Milella et al. 2011 [47], Hartzfeld et al. 2002 [48], Perez-Jimenez and Saura-Calixto 2005 [49] with some modification according to Ronda et al. 2015 [50]. Two solvents were prepared: Solvent A (HCl/methanol/water concentrate—1:80:10; *v*/*v*/*v*) and Solvent B (methanol/sulphuric acid—10:1 *v*/*v*), for the extraction of soluble and insoluble polyphenols, respectively. A total of 0.500 g of each flour sample was weighed into 15 mL centrifuge tubes and 4 mL of Solvent A was added. The tubes were agitated at laboratory orbital shaker (WU4, Premed, Marki, Poland) at room temperature for 2 h, and then centrifuged at 3500 g for 10 min (MPW-350, MPW, Warszawa, Poland) and the supernatant was collected. The extraction was repeated, and the supernatants were combined and used for soluble polyphenol content (SPC) determination. To extract the insoluble polyphenols, 5 mL of solution B was added to the residue obtained after the second centrifugation and the tubes were agitated (Shaking Incubators 3033 with Orbital Motion, GFL, Burgwedel, Germany) at 85 °C for 24 h. After centrifugation at 3500× *g* for 10 min (MPW-350, MPW, Warszawa, Poland) the supernatant containing insoluble polyphenols was collected. For measuring the reaction effect with Folin-Ciocalteu Reagent 20 μL of a sample, 1.58 mL of distilled water and 100 μL of Folin-Ciocalteu Reagent were mixed. The mixture was left to stand for 4 min and then 300 μL of a saturated solution of Na2CO3 was added. The samples were incubated (MLL147, AJL Electronics, Kraków, Poland) at 40 °C for 30 min and the absorbance at 765 nm was measured (UV-Vis Ultrospec 2000, Pharmacia Biotech, Piscataway, NJ, USA). The results were expressed as gallic acid equivalent (GAE) per gram of sample dry matter. The tests were carried out in triplicate.

### 3.9. Antioxidant Activity using DPPH Scavenging Assay 

The antioxidant capacity of the samples was determined by the DPPH method. The dual extraction system using solvents of different polarity was employed. Extracts using a low polarity solvent (HCl/methanol/water concentrate—1:80:10; *v*/*v*/*v*) were obtained in procedure described in 3.8, for Solvent A. Extracts using high polarity solvent as proposed by Collar et al. 2014 [51] were prepared as follows. A methanol/water solution (50% *v*/*v*—Solvent I), and an acetone/water solution (50% *v*/*v*—Solvent II) were prepared, respectively. A total of 2.00 g of each sample of flour was weighed into 50 mL centrifuge tubes and 20 mL of Solvent I was added to each tube and shook at laboratory orbital shaker (WU4, Premed, Marki Poland) at RT for 1 h. The tubes were centrifuged (MPW-350, MPW, Warszawa, Poland) for 10 min at 2500 g and the supernatant was separated. A total of 20 mL of Solution II was added to the residue in the tube and proceeded as previous. The supernatants were combined and adjusted by up to 50 mL with methanol. A DPPH/methanol working solution was prepared with an absorbance of 0.9 ± 0.1. 2 mL of this solution was then added to the cuvetes, together with 100 μL of the supernatant mixture and absorbance was measured at 517 nm after 30 min. The antioxidant capacity was expressed as micromole Trolox equivalent per gram of sample dry basis (μmol TE/g d.b.). 

### 3.10. Color Measurement

Color was measured using Konica Minolta CR-310 chroma meter (Ramsey, NJ, USA) connected with a Data Processor (DP-301), launched via RS232 serial port to the personal computer. To measure the color of flour, the CR-A50 accessory for granular samples measurement was used. Color parameters were taken in triplicate, while each measurement was taken as a mean of three measurements. Parameters were presented as L*, a*, b*, Chroma, and hue.

### 3.11. Statistical Analysis

Statgraphics Centurion XVI (Statpoint Technologies, Warrenton, VA, USA) was used for the analysis of variance (ANOVA) and Fisher’s least significant difference test (LSD) was applied to evaluate significant differences (*p* < 0.05) among samples. Pearson correlation among variables was also established.

## 4. Conclusions

Ultrasound treatment can be an effective method for the modulation of raw material functional characteristics. Mechanical waves introduce severe changes in the native structure of plant cells, which not only supports extraction, but also may impact hydration capacity, gel syneresis, or pigment location. Buckwheat grain underwent the ultrasound treatment, and the flour obtained from such a treated grain revealed significantly modified pasting properties and bioactive characteristics. The solid to liquid ratio during the US treatment seemed to have an essential impact on granulometry distribution, which resulted in gels’ rheology modulation. The partial extraction of bioactive compounds during the US treatment of buckwheat grains changes the profile of polyphenol content (soluble to insoluble polyphenol ratio). Ultrasound seems to be a physical treatment which has excellent potential as a rheology modifier. The ultrasound treatment application at gluten-free raw materials results in essential functional properties modification, resulting in higher stability of gels. Changes such as non-chemical allow the “clean label” exploitation of products made with the use thereof. 

## Figures and Tables

**Table 1:** Distribution of different fraction size in samples.

Sample	>200 µm	>150 µm	>106 µm	<106 µm
control	20.15 ± 1.63 ^a,b^	46.85 ± 4.03 ^b^	29.65 ± 0.64 ^b^	4.85 ± 2.19 ^a^
1:2.5	29.65 ± 0.07 ^b^	47.05 ± 3.75 ^b^	19.55 ± 6.29 ^a^	4.60 ± 2.26 ^a^
1:5	27.20 ± 7.07 ^b^	47.70 ± 0.28 ^b^	19.05 ± 7.57 ^a^	6.95 ± 0.35 ^a^
1:10	15.50 ± 1.84 ^a^	27.80 ± 0.99 ^a^	42.20 ± 2.97 ^c^	14.15 ± 0.64 ^b^

1:2.5, 1:5, 1:10-solid:liquid ratio of buckwheat grain samples ultrasound treated in water; control–sample of untreated buckwheat grain. Lower-case letters mean values are significantly different at *p* ≤ 0.05 in the column.

**Table 2:** Water Absorption Index (WAI), Water Solubility Index (WSI) and Swelling Power (SP) values of samples from different ultrasound treatments.

Sample	WAI (g/g d.b.)	WSI (g/g d.b × 100)	SP (g/g d.b.)
control	6.03 ± 0.03 ^d^	4.61 ± 0.31 ^a^	6.33 ± 0.01 ^d^
1:2.5	5.69 ± 0.00 ^b^	5.83 ± 0.11 ^b^	6.05 ± 0.01 ^b^
1:5	5.87 ± 0.01 ^c^	5.51 ± 0.16 ^b^	6.22 ± 0.01 ^c^
1:10	5.28 ± 0.04 ^a^	5.61 ± 0.18 ^b^	5.60 ± 0.04 ^a^

1:2.5, 1:5, 1:10-solid:liquid ratio of buckwheat grain samples ultrasound treated in water; control–sample of untreated buckwheat grain; d.b.–dry basis. Lower-case letters mean values significantly different at *p* ≤ 0.05 in the column.

**Table 3:** Pasting curve parameters.

Sample	PV (mPa × s)	HV (mPa × s)	FV (mPa × s)	BD (mPa × s)	SB (mPa × s)
control	2798.0 ±66.5 ^a^	2701.5 ± 50.2 ^b^	4958.0 ± 63.6 ^b^	96.5 ± 16.3 ^a^	2256.5 ± 13.4 ^b^
1:2.5	2687.0 ± 114.6 ^a^	2359.0 ± 45.3 ^a^	4467.5 ± 140.7 ^a^	328.0 ± 69.3 ^b^	2108.5 ± 95.5 ^b^
1:5	3072.0 ± 36.8 ^b^	2633.5 ± 27.6 ^b^	5169.5 ± 126.6 ^b^	438.5 ± 9.2 ^c^	2536.0 ± 99.0 ^c^
1:10	2700.5 ± 31.8 ^a^	2438.0 ± 56.6 ^a^	4203.0 ± 69.3 ^a^	262.5 ± 24.7 ^b^	1765.0 ± 12.7 ^a^

1:2.5, 1:5, 1:10-solid:liquid ratio of buckwheat grain samples ultrasound treated in water; control–sample of untreated buckwheat grain. PV–peak viscosity, HV—hold viscosity, FV—final viscosity, BD—breakdown viscosity, SB—setback viscosity. Lower-case letters mean values significantly different at *p* ≤ 0.05 in the column.

**Table 4:** Pasting process data.

Sample	V @ 1 min (mPa × s)	PPV (mPa × s)	PPt (s)	PPTp (°C)
control	40.0 ± 1.4 ^c^	489.0 ± 7.1 ^a^	234.0 ± 0.0 ^c^	85.3 ± 0.0 ^c^
1:2.5	32.5 ± 0.7 ^a^	501.0 ± 11.3 ^a^	228.5 ± 0.7 ^b^	84.5 ± 0.5 ^b^
1:5	36.0 ± 1.4 ^b^	527.0 ± 15.6 ^a^	222.0 ± 0.0 ^a^	82.7 ± 0.0 ^a^
1:10	29.5 ± 0.7 ^a^	483.5 ± 31.8 ^a^	247.5 ± 0.7 ^d^	88.8 ± 0.1 ^d^

1:2.5, 1:5, 1:10—solid:liquid ratio of buckwheat grain samples ultrasound treated in water; control–sample of untreated buckwheat grain. PPV—pasting point viscosity, PPT—pasting point time, PPTp—pasting point temperature, V @ 1 min—viscosity at 1 min. Lower-case letters mean values significantly different at *p* ≤ 0.05 in the column.

**Table 5:** DPPH scavenging activity of two types of extracts obtained from grains of different ultrasound treatments.

**Sample**	**LP Antioxidant Activity** **(μmol TE/g d.b.)**		**HP Antioxidant Activity** **(μmol TE/g d.b.)**	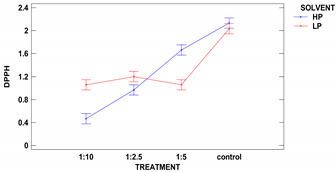
control	2.03 ± 0.05 ^b^		2.13 ± 0.00 ^d^	
1:2.5	1.20 ± 0.14 ^a^		0.97 ± 0.06 ^b^	
1:5	1.06 ± 0.14 ^a^		1.66 ± 0.06 ^c^	
1:10	1.05 ± 0.02 ^a^		0.47 ± 0.02 ^a^	
**Second Order Interactions Analysis *p* ≤ 0.05**				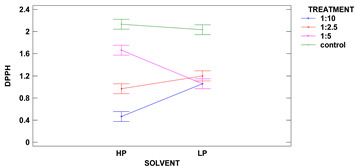
treatment		***	***	
solvent		ns	ns	
treatment × solvent		***	***	

1:2.5, 1:5, 1:10-solid:liquid ratio of buckwheat grain samples ultrasound treated in water; control–sample of untreated buckwheat grain. LP–low polarity extracts made with acidified methanol, HP–high polarity extracts made with 50% methanol and 50% acetone (both with water), d.b.–dry basis. Lower-case letters mean values significantly different at *p* ≤ 0.05 in the columns, ***—significantly different at *p* ≤ 0.001, ns–not significantly different.

**Table 6:** Effects of ultrasound treatment on color of buckwheat flour.

Sample	L*	a*	b*	C	h
control	85.35 ± 0.02 ^d^	0.92 ± 0.01 ^a^	7.76 ± 0.04 ^a^	7.81 ± 0.04 ^a^	83.35 ± 0.07 ^d^
1:2.5	84.15 ± 0.01 ^b^	1.19 ± 0.01 ^c^	7.85 ± 0.04 ^ab^	7.93 ± 0.04 ^bc^	81.50 ± 0.00 ^b^
1:5	84.76 ± 0.02 ^c^	1.12 ± 0.01 ^b^	7.83 ± 0.06 ^ab^	7.91 ± 0.05 ^ab^	81.95 ± 0.21 ^c^
1:10	82.66 ± 0.06 ^a^	1.31 ± 0.00 ^d^	7.93 ± 0.01 ^b^	8.03 ± 0.01 ^c^	80.65 ± 0.07 ^a^

1:2.5, 1:5, 1:10—solid:liquid ratio of buckwheat grain samples ultrasound treated in water; control–sample of untreated buckwheat grain. L*—lightness from black (0) to white (100), a*—green (−) to red (+), b*—blue (−) to yellow (+), C—chroma, h—hue. Lower-case letters mean significantly different at *p* < 0.05 in the column.
